# Recommendations for In Vitro and In Vivo Testing of Magnetic Nanoparticle Hyperthermia Combined with Radiation Therapy †

**DOI:** 10.3390/nano8050306

**Published:** 2018-05-06

**Authors:** Spiridon V. Spirou, Sofia A. Costa Lima, Penelope Bouziotis, Sanja Vranješ-Djurić, Eleni Κ. Efthimiadou, Anna Laurenzana, Ana Isabel Barbosa, Ignacio Garcia-Alonso, Carlton Jones, Drina Jankovic, Oliviero L. Gobbo

**Affiliations:** 1 Department of Radiology, Sismanoglio General Hospital of Attica, Sismanogliou 1, 15126 Marousi, Athens, Greece; 2 LAQV, REQUIMTE, Departamento de Ciências Químicas, Faculdade de Farmácia, Universidade do Porto, 4050-313 Porto, Portugal; slima@ff.up.pt (S.A.C.L.); up200800307@ff.up.pt (A.I.B.); 3 Institute of Nuclear & Radiological Sciences & Technology, Energy & Safety, National Center for Scientific Research “Demokritos”, Aghia Paraskevi, 15310 Athens, Greece; bouzioti@rrp.demokritos.gr; 4 “Vinča” Institute of Nuclear Sciences, University of Belgrade, 11351 Belgrade, Serbia; sanjav@vinca.rs (S.V.-D.); drinaj@vinca.rs (D.J.); 5 Inorganic Chemistry Laboratory, Chemistry Department, National and Kapodistrian University of Athens, Panepistimiopolis, 15784 Zografou, Greece; efthim@chem.chem.uoa.gr; 6 Institute of Nanoscience and Nanotechnology, NCSR Demokritos, Agia Paraskevi Attikis, 15310 Athens, Greece; 7 Department of Biomedical and Clinical Science “Mario Serio”, University of Florence, 50134 Firenze, Italy; anna.laurenzana@unifi.it; 8 Department of Surgery, Radiology & Ph.M. University of the Basque Country, E48940 Bilbao, Spain; ignacio.galonso@ehu.es; 9 NanoTherics Ltd., Studio 3, Unit 3, Silverdale Enterprise Centre Kents Lane, Newcastle under Lyme ST5 6SR, UK; carl.jones@nanotherics.com; 10 School of Pharmacy and Pharmaceutical Sciences, Panoz Institute, Trinity College Dublin, D02PN40 Dublin, Ireland

**Keywords:** animal models, biodistribution, in vitro assays, in vivo evaluation, magnetic nanoparticles, pharmacokinetics

## Abstract

Magnetic nanoparticle (MNP)-mediated hyperthermia (MH) coupled with radiation therapy (RT) is a novel approach that has the potential to overcome various practical difficulties encountered in cancer treatment. In this work, we present recommendations for the in vitro and in vivo testing and application of the two treatment techniques. These recommendations were developed by the members of Working Group 3 of COST Action TD 1402: Multifunctional Nanoparticles for Magnetic Hyperthermia and Indirect Radiation Therapy (“Radiomag”). The purpose of the recommendations is not to provide definitive answers and directions but, rather, to outline those tests and considerations that a researcher must address in order to perform in vitro and in vivo studies. The recommendations are divided into 5 parts: (a) in vitro evaluation of MNPs; (b) in vitro evaluation of MNP-cell interactions; (c) in vivo evaluation of the MNPs; (d) MH combined with RT; and (e) pharmacokinetic studies of MNPs. Synthesis and characterization of the MNPs, as well as RT protocols, are beyond the scope of this work.

## 1. Introduction

Radiation therapy (RT) is one of the main treatment approaches for cancer. It is estimated that 50–60% of all cancer patients will receive RT as part of their treatment, either alone as monotherapy or as part of a combination treatment [1,2]. Over the past 30 years, RT has undergone tremendous improvement with the introduction of imaging techniques, such as computed tomography (CT), magnetic resonance imaging (MRI), positron emission tomography (PET), the development of advanced treatment techniques, such as 3D Conformal RT, Intensity-Modulated RT [3,4,5], Image-Guided RT [6,7,8], Volumetric Modulated Arc Technique [9,10], Stereotactic Body RT [11,12,13], new modalities, such as proton therapy [14,15,16], as well as great strides in understanding tumor biology and its interaction with radiation [2,17].

Alongside these developments, the efficacy of RT can be increased by combining it with other forms of treatment, such as Hyperthermia (HT). Beginning in the 1960s, a series of in vitro experiments demonstrated that although HT by itself cannot provide curative treatment for cancer, it exhibits high synergy with RT and can significantly improve its potency [18,19,20,21]. Specifically, (a) hypoxic cells that are radio-resistant are heat-sensitive [22,23,24,25,26]; (b) cells in the S-phase that are radio-resistant are heat-sensitive [27,28,29]; (c) the low extracellular pH in tumor microenvironment reduces the efficacy of RT but increases that of HT [30,31,32,33,34]; (d) the abnormal vasculature of the tumor prevents heat from being carried away, in contrast to normal tissue, thus resulting in selective heating of the tumor [33,35,36,37,38]; and (e) within the tumor, the low blood flow in hypoxic regions results in heat being trapped [33,35,37,38].

Clinical trials, however, failed to deliver the expected results, primarily due to difficulties in adequately heating the tumor. This was especially true for deep-seated tumors. For example, a 1996 Phase III study comparing interstitial RT with and without HT for various tumor types, showed that the addition of HT did not improve treatment results. However, subsequent analysis showed that only one patient out of 86 in the combined treatment arm of the study had been adequately heated [39]. Nowadays, nanomedicine has been used to develop new ways to induce hyperthermia. For example gold nanospheres are used in photothermal therapy [40] while some iron oxide nanoparticles have been specifically designed for magnetic hyperthermia [41].

Magnetic Hyperthermia (MH) is a cutting edge technology, still in its infancy, that is based on the principle of embedding a heating source, namely magnetic nanoparticles (MNPs), into the targeted tissue/tumor and heating it by using an external alternating magnetic field [41]. Its attractiveness lies in the fact that: (a) the MNPs can, in principle, reach deep-seated tumors via the bloodstream [42,43]; (b) the location of the MNPs can be determined using various imaging techniques [44,45,46]; (c) the MNPs can also act as temperature probes, providing detailed 3D information about the temperature distribution in the tumor [47,48]; (d) the MNPs can be functionalized with specific ligands in order to improve targeting [45,49,50]; (e) the MNPs can be conjugated with chemotherapeutic drugs to increase the efficacy of magnetic hyperthermia [51]; (f) the technique is minimally invasive, requiring only a simple injection; and (g) the procedure is rather comfortable since the patient is not required to wear special suits or be immersed in a thermal bath [52].

To date, MNP-mediated MH combined with RT has only been used to treat recurrent glioblastoma multiforme, a very aggressive type of brain tumor [53]. With the proviso that this was a non-randomized, single-arm study, and therefore the results cannot be considered to be definitive, patient survival increased by several months compared with traditional patterns.

The purpose of this review is not to describe MNPs, their properties, characteristics and potential uses for MH but, rather, to outline the steps that one needs to take in order to apply the MNPs in a pre-clinical setting. These steps include in vitro evaluation of the MNPs themselves and of MNP-cell interactions, in vivo evaluation, considerations for the combination of MH and RT, and pharmacokinetic and biodistribution studies. For this reason, we have chosen to format the paper as a step-by-step, how-to guide.

## 2. Sample Preparation for In Vitro Evaluation

In vitro evaluation is of paramount importance for further in vivo investigation of MNPs. Samples revealing marginal or no toxicity via in vitro tests can then move on to in vivo studies. Nevertheless, it is not unlikely that in vitro and in vivo results are contradictory. This may be attributed to in vivo body functions and processes. The following recommendations are suggested to make a safe environment for people starting to work in this new field and to facilitate pre-clinical work using HT in combination with RT for the treatment of cancer.

Basic requirements for biological in vitro assessment include:

### 2.1. Colloidal Stability

One of the most used analytical techniques to evaluate the size of nanoparticles is Dynamic Light Scattering (DLS), and it has been extensively used since it is not time consuming, it is non-invasive and very sensitive to the presence of small aggregates. This feature can be very helpful when testing colloidal stability of MNPs [54]. In order to enhance colloidal stability, MNPs are usually functionalized with various biocompatible compounds (e.g., citrate, phosphate, chitosan, polyethylene glycol, albumin), since uncoated MNPs tend to flocculate after their introduction to physiologically relevant fluids. To achieve a better understanding of their behavior when in contact with biorelevant media, the determination of size and zeta potential as a function of time in different media (culture media, phosphate buffer, in the absence or presence of Fetal Bovine Serum (FBS)) should be assessed [55]. Indeed, studying interactions between MNPs and plasma and blood platelets for example, can be highly relevant when looking at their potential clinical application. It has previously been demonstrated that some types of NPs may lead to platelet aggregation and therefore to thrombus formation [56]. However, it is important to highlight that this detrimental effect can be also reverted, for example, by introducing PEGylation for improving their compatibility [57]. Another issue to take into account is the influence of MNP concentration on the size and zeta potential, which should have a negative value to avoid cytotoxicity. An approximate value of −30 mV should be taken as the reference to assure colloidal stability. Also, correct DLS measurement relies on the concentration of the sample which, in turn, is based on the type of the materials and their size. For a correct characterization, an optimal concentration should be correctly determined.

### 2.2. Sterilization of the Aqueous MNP Solution

There are several sterilization methods that can be applied to nanomaterials, such as filtration [58,59], autoclaving [60], irradiation [61], the gas plasma method [62], and others [62]. Endotoxin contamination is a general problem in clinical use, not specific to nanomaterials. The methods described in this paragraph are used in general for the removal or destruction of microbial contamination. In addition, in the case of nanomaterials, it is crucial to investigate how these techniques may affect the physico-chemical and biological properties of the nanomaterials, such as stability and toxicity [63] by re-characterization and evaluation.

#### Sterilization of the Aqueous MNP Solution Using Filtration

Once in aqueous solution, MNPs tend to be prone to contamination. The process of sterilization by filtration is a commonly used technique to exclude bacteria, mold and yeast. Filtration using a filter or membrane with a pore size of 0.22 μm or smaller is adequate to remove contaminants of biological origin. This process does not affect the characteristics of the MNPs, since their size is smaller than that of the pore used. Azide is a very toxic substance used to prevent contamination. It is added to the incubator water at a very low concentration, which does not affect the MNPs.

### 2.3. MNP Samples Should Be Tested for Endotoxin and Used When Endotoxin-Free

Endotoxins are membrane constituents in most Gram-negative bacteria that can be identified by immune cells as pathogen-associated molecules. If these compounds are found in nanoparticles suspension, in vitro results might represent the effect of the contamination and hamper the analysis of toxicological and inflammatory outcomes associated to MNPs. The FDA recommends validating the alternative methods for each product to detect endotoxin. This problem is very common in studies of nanomaterials and it is not easy to eradicate. A common method for endotoxin detection is the Limulus amoebocyte lysate (LAL) assay, but this type of bioassay is not adequate for all types of MNPs. Because of the difficulties associated with endotoxin removal, preventing contamination during MNP synthesis would be the best way for obtaining endotoxin-free particles. This can be achieved by using only endotoxin-free reagents including water and buffers, instruments and glassware during the synthesis process [64]. Of particular importance is the reaction solvent. In the case of water, the pH (extremely acidic or basic) and temperature (high or low) are crucial parameters that must be taken into consideration. Organic solvents reduce the probability of contamination due to their toxicity.

To store the MNPs for later use, it is better to keep them in the solid phase and apply one of the techniques suggested shortly before preparing the suspension. MNPs in the solid phase can be sterilized by exposing them to ultraviolet radiation (UV).

### 2.4. A High Working Concentration of MNPs Is Recommended

The importance of a high working concentration (>10 mg mL^−1^) is suggested to widen the tested conditions. Higher concentrations of MNPs broaden the application of different electric and magnetic fields according to the intended application. To determine the appropriate concentration of MNPs per cell for HT, the MNPs should be evaluated at different concentrations and under different frequencies and power of the magnetic field. All these parameters are related to the shape, size and charge of the MNPs.

### 2.5. The Formation of Protein Corona in the Presence of Fetal Bovine Serum

When MNPs are in the blood stream, they interact with albumin, creating a layer on their surface called the protein-corona layer. It is important to know how the MNP size affects colloidal stability and the internalization into the cells. The protein corona formed can reduce the toxicity of MNPs, however the mechanisms involved are still under investigation [65]. The behavior of MNPs in the presence of albumin can be investigated in vitro, using Fetal Bovine Serum.

### 2.6. Sonication and Vortexing to Assure Homogeneity of the MNPs

Since colloidal stability is a priority parameter in sample preparation, it is also necessary to avoid all signs of aggregation. A reproducible concentration-effect response can be obtained only with a homogeneous solution, which in turn is the result of adequate sonication and vortexing of the samples under investigation.

### 2.7. Cells Should Be Confluent but Not Overgrown

In order to proceed to quality cell culture assays, it is important to maintain the morphology of the chosen cell line. Careful observation of the cells under the microscope upon handling allows the detection of signs of contamination or detachment and differences in their shape and appearance. To confirm the handling of a healthy cell line, cells should reach confluence, but never exceed that state for more than 24 h, to avoid deterioration.

### 2.8. Properties of the MNPs

**Figure 1:**
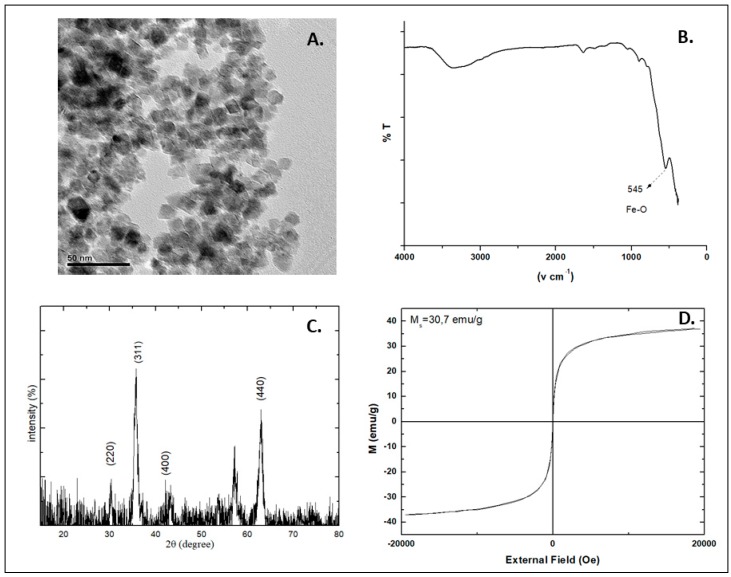
Typical transmission electron microscopy (TEM) (**A**), fourier transformed-infrared spectroscopy (FT-IR) (**B**), X-ray powder diffraction (XRD) (**C**) and vibrating sample magnetometer (VSM) (**D**) characterization of MNPs (maghemite-magnetite mixture). (Unpublished data).

The properties of the MNPs should be determined alone, in vitro and in vivo, before and after application of Alternating Magnetic Field (AMF) as that may affect their structure and composition. An example of transmission electron microscopy (TEM), fourier transformed-infrared spectroscopy (FT-IR), X-ray powder diffraction (XRD) and vibrating sample magnetometer (VSM) characterization is presented in Figure 1. TEM imaging shows that the MNPs have spherical shapes and their size is about 10 ± 2 nm (Figure 1A). The FT-IR spectrum of the MNPs has a peak at 545 cm^−1^, which is characteristic of the Fe-O bond vibration (Figure 1B). X-ray diffraction (XRD) clearly shows the inverse cubic spinel structure of Fe_3_O_4_ (Figure 1C). Positions of the diffraction peaks (220), (311), (400), (422), (511), (440) and (533) match well enough with standard XRD patterns for bulk magnetite, indicating that the phase of the nanoparticles is pure magnetite (Fe_3_O_4_). The MNPs are well crystallized and their size can be estimated using Scherer’s equation [66]. This equation uses the reference peak width at angle 2h = 35.7, where *k* is the X-ray wavelength (1.5418 Å); *b* is the width of the XRD peak at half height, with a value of 0.446 and is calculated by Lorentzian fitting of the most intense peak (311). *K* is a shape factor, about 0.9 for magnetite. The estimated size of the MNPs is about 8 nm. The magnetization curve of the MNPs is presented in Figure 1D, where the saturation magnetization (Ms) is 37.05 emu/g. The high Ms, and the small size indicate that the MNPs may present super-paramagnetic properties. Even though the saturation magnetization value is lower than that of bulk magnetite (90 emu/g), it is sufficient for magnetic hyperthermia applications.

## 3. In Vitro Evaluation of MNP-Cell Interactions

The physico-chemical properties of MNPs facilitate cellular interactions that result in their biological accumulation. Diverse cellular effects have already been described involving decrease in cell viability, plasmatic membrane disruption, mitochondrial alterations and even cell cycle arrest upon exposure to MNPs. The nature of the nanoparticle (e.g., core type and coating material), applied dose, exposure time, and cell and culture conditions are some of the factors that influence the MNP-cell interaction [67]. Additionally, the choice of assay to evaluate this process may affect the outcome, resulting in the controversial studies found in the literature. The design and experimental setup of each assay should be carefully considered [68]. In this section, we discuss the relevant aspects of the most commonly used assays to evaluate MNP-cell interactions.

### 3.1. Toxicity

General toxicity tests, aimed mainly at determining the biological activity of NPs, can be carried out on many cell types (e.g., fibroblasts, HeLa and hepatoma cells), grown either in suspension (HL60, K562) or in monolayers (MCF-7, U87MG) and cultured in 75-cm^2^ flasks (see Table 1). A number of parameters, including vital staining, cytosolic enzyme release, cell growth and cloning efficiency, are used as end-points to measure toxicity. The most common and simplest methods to investigate cell viability are colorimetric assays like 3-(4,5-dimethylthiazolyl-2)-2,5-diphenyltetrazolium bromide (MTT), sodium 3′-[1-(phenylaminocarbonyl)-3,4-tetrazolium]-bis (4-methoxy-6-nitro) benzene sulfonic acid hydrate (XTT) and others [69,70].

In a typical setup for these assays, cells (100 μL, 1 × 10^5^ cells/mL) are seeded in 96-well flat-bottomed plates, although their concentration may vary depending on the exposure time to toxicants. The cells are incubated at 37 °C with 5% CO_2_ for 24, 72, 120 and 168 h (for the 120- and 168-h incubations, the medium is replaced at 72 h with fresh medium containing the appropriate concentrations of MNPs). The cells are washed three times with PBS and replaced with fresh medium with the colorimetric substances (100 μL). The absorbance is measured, and the viability of the cells expressed as a function of concentration and exposure time.

A summary of cytotoxicity studies of MNPs is shown in Table 1 [71].

### 3.2. Cell Viability

In an effort to determine cell viability, a number of assays correlated with either membrane integrity, metabolic activity or functional capabilities have been developed. Exposure of cells to MNPs can lead to plasmatic membrane disruption, allowing the release of cellular molecules, such as lactate dehydrogenase (LDH), or the entry of molecules able to intercalate with nucleic acids(e.g., propidium iodide, PI) [72]. Tetrazolium salts (e.g., MTT and XTT) are commonly used to determine cell viability, as mitochondrial activity reduces these subtracts into formazan crystals [73,74,75,76]. The spectrophotometric detection of these crystals between 450 and 570 nm might be hampered by the interference of the MNP absorbance; thus, special care should be taken when selecting the assay conditions. Alternatively, cell viability can be determined through fluorescence or luminescence using an adenosine triphosphate (ATP)-based assay, that quantifies a product generated upon phosphorylation of glycerol; or a live/dead assay, based on the simultaneous determination of live and dead cells (measures the intracellular esterase activity and plasma membrane integrity). The Resazurin cell viability assay is also a reliable, sensitive and easy-to-use fluorescent assay that detects cellular metabolic activity as resazurin (7-Hydroxy3H-phenoxazin-3-one 10-oxide), a non-fluorescent dye, is irreversibly reduced to the highly red fluorescent resorufin by dehydrogenase enzymes in metabolically active cells. The Trypan Blue dye exclusion test is commonly used to determine the number of viable cells present in a cell suspension, as only live cells with intact membranes exclude the entry of the dye. Upon optical microscope examination, a viable cell will have a clear cytoplasm, whereas a nonviable cell will have a blue cytoplasm.

### 3.3. Cell Proliferation

Cell proliferation assays are mainly designed based on three concepts: analysis of metabolic activity (previously described), measurement of the rate of DNA replication and, less commonly, recognition of cell surface antigen [77]. The rate of DNA replication can be analyzed by using radioactive (e.g., 3H-thymidine) or labelled nucleotide analogues (e.g., 5-bromo-2′-deoxyuridine, BrdU). BrdU can be incorporated into DNA during proliferation and assessed by flow cytometry or immunohistochemistry after addition of fluorescently-labelled antibodies specific for BrdU. Alternatively, cells can be labelled with carboxyfluoresceinsuccinimidyl ester (CFSE), which is incorporated into DNA during proliferation and is measured using a flow cytometer. The signal is proportional to the quantity of CFSE incorporated into cells.

### 3.4. MNP Cellular Uptake Studies

Assessment of MNP-cell interactions should allow single cell analysis to discriminate surface bound and internalized nanoparticles. Usually, cellular uptake studies involve imaging techniques and flow cytometry methods [78]. Fluorescence microscopy-based techniques allow one to distinguish the nanoparticle signal inside the cells from the different cellular constituents, as different labeling methods can be applied. However, to reach a resolution in the nanometer range electron microscopy represents a more suitable technique, allowing the localization of nanoparticles at the ultrastructural cell level. Consequently, transmission electron and scanning electron microscopy techniques play an important role in the evaluation of the cellular framework, including organelles and membrane structures [79]. It is important to note that electron microscopy only gives a static image of the sample, requiring an immobilization step using a resin or cryogenic conditions. The flow cytometry-based approach allows higher sample throughput and provides only qualitative information on cell-associated nanoparticles. Analysis with flow cytometry usually requires fluorescent nanoparticles, but it is also possible to analyze cellular association of non-fluorescent nanoparticles, taking advantage of the side scatter function of flow cytometry [80] that identifies the cellular granularity. To distinguish both surface-bound and internalized nanoparticles, the previously described imaging techniques are recommended.

### 3.5. Other Complementary Assays

The Caspase-3/7 green assay enables the flow cytometric detection of activated caspase-3 and caspase-7 in apoptotic cells. Apoptosis can be evaluated by annexin V, an intracellular protein that binds to phosphatidylserine in a calcium-dependent manner. Fluorochrome-labeled annexin V can then be used to specifically target and identify apoptotic cells. To help distinguish between the necrotic and apoptotic cells it is recommended to use PI or 7-amino-actinomycin D (7-AAD). Early apoptotic cells will exclude PI or 7-AAD, while late-stage apoptotic cells will stain positively, due to the passage of these dyes into the nucleus, where they bind to DNA. DNA fragmentation can be detected via the TUNEL (Terminal deoxynucleotidyl transferase dUTP nick end labeling) assay, as the DNA strand breaks are detected by enzymatically labeling the free 3′-OH termini with modified nucleotides. These new DNA ends are typically localized in morphologically identifiable nuclei and apoptotic bodies. In contrast, normal or proliferative nuclei, which have relatively insignificant numbers of DNA 3′-OH ends, usually do not stain with the kit.

## 4. Sample Preparation for In Vivo Evaluation

With regard to MNP preparation, a pH solution range of 7.2 ± 0.6 should be considered mandatory. Indeed, this neutral pH should avoid damaging the veins or inducing pain to the animals. To prevent or reduce vascular complications, the osmolarity of the MNP solution also has to be considered [81]. A very small number of in vivo experiments have tested the direct cytotoxic effects on vascular endothelial cells due to pH and/or osmolarity changes, after intravenous administration of drugs [81]. The viscosity and the rate of infusion are also very important to consider when administering MNPs through vascular vessels [82].

**Figure 2:**
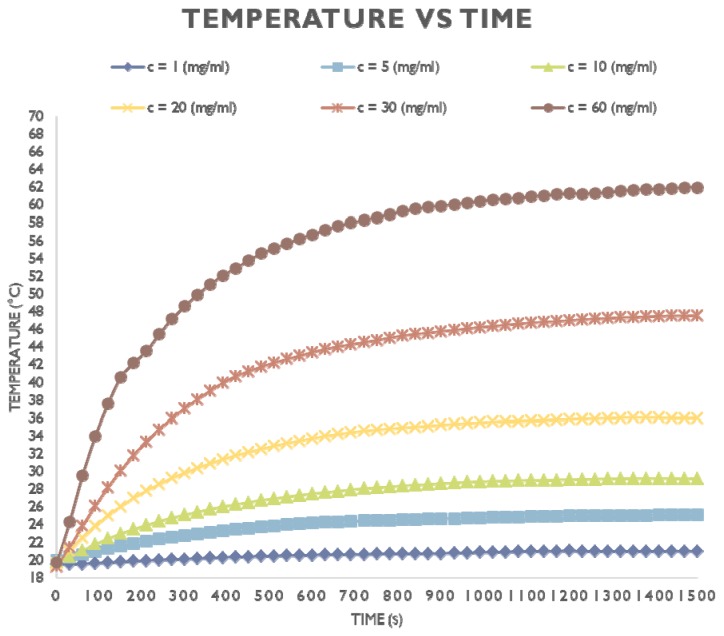
Example of temperature response as a function of time and concentration of the MNPs (maghemite-magnetite mixture) under AMF. The temperature was measured with the Ambrell EasyHeat system (Magnetic Field Amplitude H0 = 30.1 kA/m, Frequency f = 275.0 kHz) using a Yokogawa fiber-optic system with Tpmeter3 software. The optical fiber was immersed in the magnetic fluid, so that the tip of the fiber would coincide with the central point of the magnetic fluid volume. A time interval of ~5 min was allowed before switching on the magnetic field, so that the system would reach the thermal equilibrium state. The desirable magnetic field could be achieved by adjusting the coil current via the EasyHeat system. A time step of 30 s was selected between consecutive temperature recordings and the total heating process lasted 1500 s. Subsequently, the field was switched off, but the data acquisition process was continued, in order to obtain the cooling phase of the fluid. (Unpublished data).

MNPs should be tested alone in a magnetic field, and a frequency response profile should be determined to optimize the MNP heating rates and establish the appropriate field/frequency combination to set up the most favorable concentration for inducing HT in vitro and in vivo (dose response vs. time). Then, a dose-response curve should be constructed, if possible (Figure 2). It is possible that the MNPs themselves may be affected by the AMF and/or temperature changes [83]. Therefore, the properties of the MNPs [84] should be checked again after HT application (e.g., size by DLS, shape by TEM). Indeed, the temperature at the surface of the MNPs could be different from the surrounding environment due to heat transfer mechanisms in close proximity to the MNPs [85]. Yu and colleagues [86] have described a method based on the temperature-dependent thermodynamics of the hybridization and denaturation of double-stranded DNA to evaluate the local temperature of different MNPs during HT.

The delivery strategy has to be decided for the in vivo evaluation. Indeed, a systemic injection of MNPs into the tumor does not face the same problems that a direct injection does. Recently, direct injection of MNPs has been approved by the European Medicines Agency (EMA) for the treatment of primary or recurrent glioblastoma multiforme, a lethal brain tumor with limited treatment options [53]. Systemic injection has the advantage that MNPs reach the tumor in a more uniform way (uniform binding), which results in improved internalization [87]. However, in the blood stream, MNPs would be subject to opsonization (i.e., the process by which an exogenous molecule is tagged for destruction by phagocytosis) [45] and captured by the reticuloendothelial system (RES) [88]. To avoid these problems, MNPs are coated with surfactants and polymers [45,88].

In order to reach the targeted diseased area, MNPs have to be designed not only to avoid uptakes but also to effectively penetrate into the tumor interstitium and to be taken up by cancer cells. This introduces the concept of targeting strategies [89]. The two main strategies are passive targeting and active targeting [45]. Passive targeting takes advantage of the abnormal vessels created by cancer-induced angiogenesis, which have fissures through which MNPs may accumulate in the tumor [45]. The success of passive targeting also depends on different physicochemical properties of the MNPs such as particle size, surface charge and hydrophobicity [90]. Moreover, solid tumors show the enhanced permeability and retention (EPR) effect for MNPs of appropriate size. The EPR effect is due to defective vasculature [91] and deficient lymphatic drainage system in solid tumors, which increase the pore size in the endothelial wall [90].

Active targeting relies on magnetic guidance of the MNPs using an external magnet, or through interaction mediated by targeting ligands (i.e., antibodies, peptides) as presented with NPs [45,92]. An external magnetic field can attract and maintain MNPs into the area of interest. Pre-clinical and clinical trials have produced very consistent results in cancer treatments [93,94,95]. Another way to increase accumulation of MNPs in the tumor is to use biotargeting agents that specifically bind to tumor cells. Yet another interesting approach to increase the concentration of MNPs in the tumor tissue is to attach specific binding epitopes to the MNPs. For example, the RGD peptide (Arg-Gly-Asp) is recognized by integrins expressed on the membrane of both colorectal carcinoma cells and the endothelial cells of newly formed vessels in the tumor [96,97,98]. Thus, the identification of specific integrins in the tumor models is the key to developing more selective MNPs [99].

## 5. Magnetic Hyperthermia Combined with Radiation Therapy

Before beginning any pre-clinical experiments, one should have a clear understanding of the physics, biology, radiobiology and physiology of the animal, the tumor, HT and RT. Several references are provided in the Introduction. These can be used as a starting point for further research.

As with all experiments, a pre-clinical study should be carefully designed and all the relevant factors that may affect the results should be considered. Below some of the more important ones regarding MNP-mediated HT are discussed. We focus on HT because in contrast, RT technology, equipment, algorithms, treatment planning and analysis software, quality assurance and procedures are well-established and mature.

### 5.1. Animal Physiology

Understanding animal physiology is the key to a successful experiment. For example, one should not forget that core body temperatures will fall significantly under anesthesia, so it is mandatory to use a rectal probe to monitor this parameter and to artificially maintain the physiological temperature of the animal during treatment [100,101,102] by placing, for example, the animal on a heated pad during experimentation and postoperative period. Otherwise, the need of heating to achieve effective hyperthermia would be unnecessarily increased. The administration of “foreign materials” (such as MNPs), which will probably be affected by the immune system, also has to be taken under consideration. Therefore, the use of immunocompromised animals may modify the outcomes of these experiments.

### 5.2. Modelling and Simulations

Both MH and RT are physical processes with biological effects. As such, they naturally lead to modelling and simulations. For example, physics modelling software can be used to estimate the quantity and distribution of MNPs required to achieve the desired temperature in the tumor, under different technical parameters, e.g., AMF frequency and strength, and physiological conditions, e.g., blood flow. In silico, biology software can be used to assess the effects of the two treatments [103].

### 5.3. Tumor Models

The tumor animal model should be considered before designing the HT treatments. Its characteristics should mimic as closely as possible the vascularization pattern of the real human tumor. Vascularization is very relevant because of its influence on the delivery of MNPs into the tumor and dissipation of heat induced by MNPs. For example, in the Dunning model [104] of prostate cancer (an established rat model developed to study prostate cancer progression), orthotopically-implanted tumors display a more rapid growth and a higher resistance to HT than heterotopic tumors, an issue which is possibly related to higher perfusion [105]. Direct implants of colorectal cancer cells in the rat liver develop their vascularization from the arterial vessels and do not receive blood from the portal vein [106]. However, tumors induced by subcutaneous tumor cell inoculation develop less stroma than their correspondent orthotopic tumors [107]. The sites of tumor implantation are also important for the experiments when direct administration of MNPs into the tumor tissue is chosen. Though imaging techniques such as US and MRI can be used to track the needle inside the animals, there are fewer complications when the tumors are easily accessible.

### 5.4. MNP Administration

Ideally MNPs should be administered by systemic injection [108] (e.g., intravenous or intraperitoneal administrations), as this is a simple and safe procedure. However, at present, this approach seems to have a low success rate in the accumulation of MNPs in the tumor [51]. In animal models, orientation has been attempted by systemically-administered MNPs using external magnets [109] or biotargeting agents (see Section 4). Alternatively, MNP administration could be realized by direct injection into the tumor [53], intraarterially [110] or by MNP vehiculization near the tumor using a catheter, which is a routine procedure in cancer therapy [111]. Several consecutive injections can also be considered as a way to achieve a minimal concentration of MNPs in the tumor tissue. In fact, this approach has been tried in some clinical settings. Direct injection is also preferred in an attempt to avoid the accumulation of MNPs in the main organs of the reticuloendothelial system (i.e., liver and spleen), which could lead to side effects with HT (see Section 5.6 Treatment).

### 5.5. Thermal Dosimetry

A critical issue in HT is how to measure the temperature distribution in the tumor and surrounding tissue in real time. Temperature probes, such as optical fibers, are direct and accurate, but invasive. Therefore, they run the risk of spreading the disease and, practically, only a limited number of them can be used. Infrared imaging provides 2D measurements but is indirect since it measures the skin rather than the tumor temperature. MRI can provide 3D temperature distributions, along with other information that can be used to plan, improve and assess the heating process as well as the ability of the tumor to be heated [48,112]. However, the presence of MNPs interferes with MRI thermometry [113]. Moreover, the availability of MRI units may be a problem, especially for pre-clinical experiments. MRI units are generally found in hospitals, so using them for laboratory animals may run into legal or regulatory issues, scheduling and disinfecting problems etc. Diffuse Optical Spectroscopic Imaging, employing low power near-infrared light, has been used in clinical settings to assess deep tissue temperature in breast cancer [114].

From the temperature map, various quantities that describe the heating achieved can be calculated. An often-quoted one is CEM43T90, which is the cumulative equivalent minutes at 43 °C covering 90% of the tumor. Although CEM43T90 is a single number for describing a spatially and temporally varying temperature distribution [115,116], it has been shown to correlate well with clinical outcomes [117,118,119,120].

### 5.6. Treatment

The choice of treatment sequence should take into account the relevant radiobiological, physiological and technical considerations, and may have a significant effect on the results. The synergy between the two treatments appears to be maximal when they are administered simultaneously [36,121,122]. However, the duration of the synergy and the optimal sequence will probably depend on the cell type. For example, for Chinese hamster ovary cells (HA-1) radiosensitization was much higher when heating preceded irradiation, but for mouse mammary sarcoma cells (EMT-6) the opposite was true [123]. In HA-1 cells the synergistic effect lasted about 20 min [124], whereas in C3H mammary carcinoma and its surrounding skin, the effect lasted more than one hour [18]. Interestingly, in the C3H mammary carcinoma study, radiosensitization was high for both the tumor and normal skin when HT and RT were given simultaneously, but a differential radiosensitization of tumor vs. normal skin was observed when there was an interval between the two treatments, especially when HT was given 4–24 h after radiation.

In a typical HT session, the entire animal is placed inside the heating coil. Thus, all the MNPs are excited, regardless of whether they are in the tumor or not. In contrast to HT, sophisticated commercial and development small animal irradiation devices that can perform stereotactic irradiation, image guidance, Monte Carlo dose calculation and proton irradiation are available [125,126,127,128]. These specialized units can deliver highly conformal dose distributions, with sub-millimeter precision, limiting the high-dose region to the tumor and sparing surrounding normal tissue. However, they are not widely available. Yet, even in the case where irradiation is performed using fixed radioactive sources, it is possible to reduce the exposed area by collimating the field.

### 5.7. Blood Flow

Heating causes the blood vessels to dilate, increases blood flow and enhances permeability through the vessel walls. These, in turn, affect heat dissipation and tumor oxygenation and the microenvironment. The effect is non-uniform and depends on the temperature, heating pattern, treatment sequencing as well as the tumor characteristics, such as its size [33,121,129,130,131]. In the case of MNP-mediated HT, this may affect the MNP distribution and/or penetration in the tumor.

### 5.8. Thermotolerance

Heating represents a stress situation for cells and, at hyperthermic temperatures (max = 45 °C [132]), they respond by expressing Heat Shock Proteins (HSP) that protect the cell from further damage [133,134,135]. This mechanism is not present at the very high temperatures reached in thermal ablation. HSPs function as chaperones within the cell and protect cells from thermal damage by stabilizing unfolded proteins to prevent aggregation. Although a lot remains to be elucidated regarding HSPs, such as their role in cancer and the immune response [136,137,138], the practical result is that cells develop thermotolerance, which may persist for 72 to 120 h [139]. Thus, HT is typically applied once or twice a week in the clinic, whereas RT is applied daily.

### 5.9. Heating Profile

Radiosensitization depends on the heating rate and pattern, i.e., whether it is continuous at a given temperature, interrupted or complex, consisting of successive heating at different temperatures. For example, heating first at a lower temperature and then at a higher one (*step-up heating*) resulted in radiosensitization that was marginally better than heating at a single temperature. The converse pattern (*step-down heating*) was slightly better overall, with the added characteristic that the differential sensitization between tumor and skin was increased [121,140,141,142].

### 5.10. Study Endpoints

The endpoints of the preclinical experiments should be carefully defined before the study begins, as these may affect the design of the study. Complete Response (CR), which means that all detectable tumor has disappeared, is often used in clinical studies [143,144,145]. Another important and practical endpoint is cancer relapse. Therefore, one could plan the preclinical protocol to test the efficacy of HT + RT in the primary tumor and to investigate the absence or presence of relapse after treatment. It may also be considered, in those models in which metastases develop from the primary induced tumor, whether metastases are delayed or completely avoided by MNP treatment.

In addition, one should look at biochemical and/or histological markers to assess the effects of HT. For example, tumor histological analysis could be done before and after treatment to assess whether HT caused differentiation in the tumor profile. Or, since HSP90 [146] and the protein kinase B/AKT (PKB/AKT) pathway are both inhibited after treatment with RT + HT, their expression levels in tumor mass could be analyzed. Another interesting point is targeting cancer stem-like cells, which are thought to be responsible for clinical relapses and are known to be extremely radioresistant. HT is being studied as a means to induce radiosensitization of these cells [147].

### 5.11. Toxicity

Although the in vivo toxicity of the MNPs themselves must be determined and found acceptable before the pre-clinical study, it is not clear that the same level of toxicity will hold when HT and RT are applied. For example, in the presence of an alternating magnetic field, MNPs induce heat that can be dissipated by the blood flow (heat transfer phenomenon), reducing the efficacy of HT [148]. To compensate this heat loss, the power of the alternating magnetic field and exposition time can be increased to improve the efficacy of HT which can damage healthy cells. Therefore, in vivo toxicity under application of HT and RT should also be investigated, e.g., by determination of biochemical markers of liver function [e.g., gamma-glutamyl transferase (γ-GT), serum glutamic-oxaloacetic transaminase (SGOT), alkaline phosphatase (ALP)], histological examination of different organs, pharmacokinetic and biodistribution studies by liquid chromatography-tandem mass spectrometry (LCMS/MS), γ-imaging, MRI, CT, etc. In any case, iron oxide nanoparticles have been found to be safe, as iron is biocompatible and degradable by human metabolism [149].

### 5.12. Treatment Evaluation

Long-term survival should be monitored for at least 60 days, since the growth rate of recurrent tumors should not be different from that of the original tumor. In addition to determining whether the treated tumors retained their tumorigenic properties after RT + HT treatment, tumors could be digested 48 h after treatment and transplanted as single cells in limiting dilution into syngeneic recipients [150,151]. Histological analysis of the newly formed tumor mass could then be performed. The efficacy of RT + HT treatment could also be evaluated using the non-invasive MRI technique. Indeed, based on their MRI contrast agent properties, MNPs could help to image tumors when they are localized in the diseased site. MRI is, therefore, a perfect imaging technique to monitor changes of the tumor volume following anticancer treatments. Together, these data should suggest that RT + HT decreases the tumorigenicity of residual tumor cells and that the surviving cells form tumors that have pathologic signs of histological differentiation with a less aggressive phenotype than either the mock-treated tumors or the tumors treated with sub lethal doses of radiation.

Though limited success could be achieved in animal tumor models, any rate of success may be clinically relevant as this procedure could be applied after major surgical removal of the tumor in order to treat remnant tumor cells in the affected organ. Thus, it should be advised that when seeing some degree of tumor reduction, new experiments with micrometastasis models would be of great interest.

At this stage, some histological samples from control animals (without HT and RT) could be used to determine the internalization of MNPs into the cells. Whether the MNPs are inside or outside the cells is very important and may differ from in vitro experiments. For example, PEG-functionalized mesoporous silica-coated MNPs have a better heating performance when outside the cells [152]. TEM and Perl’s staining are the most suitable techniques for such investigations.

### 5.13. Analysis of Results

One should be careful when interpreting and analyzing the results from the combined treatment. The interaction between radiation alone, without HT, and the animal or human is complex and multifaceted. For example, radiation can simultaneously have an immunosuppressive as well as an immunostimulant effect [153,154,155,156]. Which of the two dominates depends on many factors, including dose, fractionation, and tumor type [157,158,159,160]. The addition of HT is an extra layer of complexity, which makes it more difficult to fully understand the mechanisms that lead to cancer cell death.

## 6. Recommended Method for Pharmacokinetic and Biodistribution Studies of MNPs

### 6.1. Pharmacokinetics

Pharmacokinetics (PK) can help to make decisions about further preclinical investigations on novel MNPs [161]. Since the chemical and physical properties of MNPs determine their in vivo fate, it is desirable to measure PK profiles early in the development process so as to use that information in MNP design and candidate prototype selection. Evaluation of absorption, distribution, metabolism and excretion (ADME) of MNPs is essential. Four factors essentially determine PK of MNPs: route of administration; particle size, shape and charge; nature of coating materials; and animal species [162,163].

The radiotracer method is particularly suited for the study of pharmacokinetic parameters of nanomaterials [164,165]. Measuring radiation from radioactive tracers attached to MNPs has been shown to be a highly sensitive and specific method that allows for accurate quantification, without limit to tissue penetration in any organ. The required radionuclide concentration is around 10^−10^ M at the site of interest. Other methods are also available to evaluate biodistribution and PK parameters of MNPs. From the innovative particle electron paramagnetic resonance (pEPR) method to the gold standard ICP-MS, various parameters, such as MNP concentration, can be evaluated in tissues and organs [166]. In the present manuscript, we will focus on the radiotracer method.

### 6.2. Biodistribution Studies

Tissue/organ/tumor accumulation is expected to be dependent on the dose and time post-injection, and hence time-course studies at different doses are important for full characterization. Tissue accumulation should be determined for more than two time-points, in order to acquire as much information on the PK of the injected MNPs as possible, which is crucial for developing design rules.

**Figure 3:**
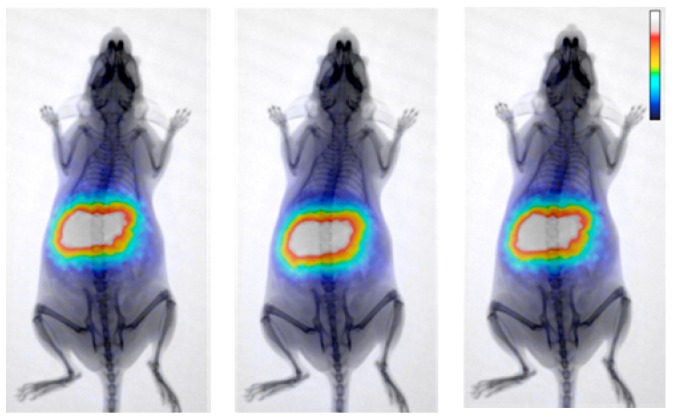
Cumulative positron emission tomography (PET) / X-radiation (X-ray) images of a normal Swiss mouse injected with ^68^Ga-Fe_3_O_4_-2,3-dicarboxypropane-1,1-diphosphonic acid (DPD) MNPs at 20, 30 and 60 min post-injection. The gradual alteration in color indicates a lower to higher number of recorded counts (Reproduced with permission from [167]).

Important biodistribution information on the MNPs under evaluation can often be obtained by radiotracer-based imaging (Figure 3) using either single-photon emission computed tomography (SPECT) or PET, depending on the available radionuclides.

If PET or SPECT are not available, the information on in vivo biodistribution of the radiolabeled MNPs can be obtained by ex vivo biodistribution studies dissecting the animal, collecting blood, organs and tissues of interest and measuring the radioactivity arising from accumulated radiolabeled MNPs by gamma counter. The animals should be sacrificed via spinal cord dislocation at specific times after injection, depending on the physicochemical characteristics of the MNPs (size, surface charge etc.) and the half-life of the radionuclide used for radiolabeling. For nanometer-sized particles, recommended time-points for assessment are 30 min, 120 min, 24 h, 48 h, 72 h after i.v. administration, to monitor their retention and excretion pattern. The following organs should be removed, weighed and measured for radioactivity: heart, lungs, liver, kidneys, spleen, stomach (emptied), intestine (emptied), muscle (hind leg), the complete left femur, and a blood sample (0.5–1 mL). All studies should be performed on at least three animals per time-point.

If beta emitters are used, before measuring radioactivity the tissues should be homogenized and diluted with water to reach identical geometry and similar probe density for bremsstrahlung measurements.

The uptake into the organs should be expressed as a percentage of the injected total radioactivity (% injected dose per organ) and per mL of blood, except for the radioactivity in the muscle, which should be estimated by assuming a muscle weight of 40% of the total body weight. Tissue uptake of the MNPs could be also expressed as % injected dose (ID) per gram (percentage of ID per gram tissue) at post-injection time. The data should be presented as mean values ± standard deviation for each group. Data should be decay-corrected for the nuclide used in each experiment.

The precise injected activity is calculated by measuring the activity of the syringe, containing the radiolabeled MNPs, before and after injection, as a significant quantity of the labeled MNPs may be adsorbed onto the plastics of the syringe.

### 6.3. Radiolabeling of MNPs, Selection of Radionuclides and Optimization of the Radiolabeling Procedure

**Figure 4:**
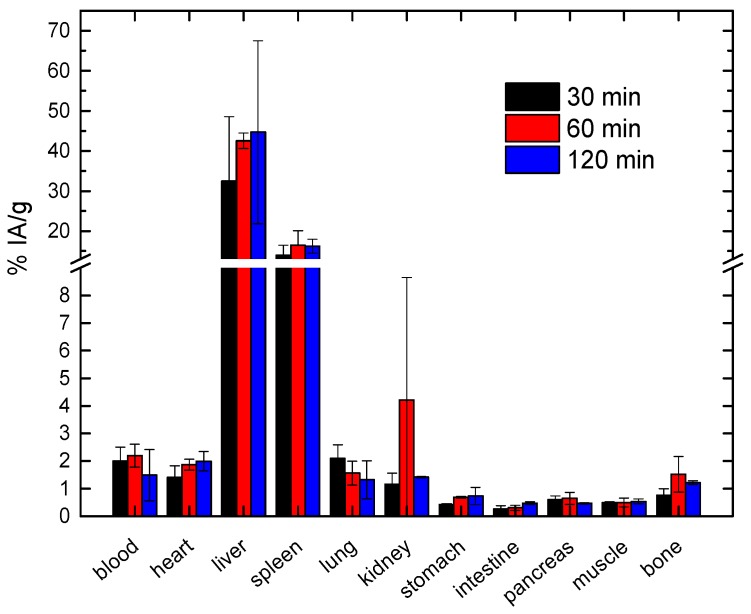
Ex vivo biodistribution study of ^68^Ga-Fe_3_O_4_-DPD MNPs in normal Swiss mice performed at 30, 60, and 120 min post injection (Reproduced with permission from [167]). Due to the relatively short half-life of ^68^Ga (t_1/2_ = 68 min), the study was performed at the designated time-points, after i.v. injection of the radiolabeled MNPs (11.11 μg MNPs/100 μL per mouse).

The selection criteria for radionuclides must be based on the physical data of the radionuclide and biological variables governing its use. The considerations for physical characteristics include the physical half-life, type of emissions, energy of the radiations, daughter products, method of production, and radionuclide purity. The biochemical aspects of the radiolabeled MNPs include tissue targeting, retention of radioactivity in the organ/tissue, in vivo stability, and toxicity. In contrast to MNP production, the radiolabeling process is time-limited and difficult because of the risk of contamination. The handling of radionuclides has to be carried out in specially designed radiochemical laboratories with controlled ventilation and air conditioning, shielded remote handling facilities, and special equipment for measuring the radioactivity of selected radionuclides. Particular attention must be paid to the potential impact of labeling on the properties of MNPs. An adapted labeling method will need to be used, to ensure that the labeling is not likely to modify the physicochemical properties of the MNPs and hence their biodistribution. It will also be necessary to ensure that this marker is not “prematurely” released from the MNPs, leading to potential confusion between monitoring of the released marker and that remaining bound to the MNPs, as in this case the radionuclide distribution will not reflect that of the MNPs. If the radiotracer method is used to follow the biodistribution (Figure 4), then the minimum activity-dose necessary to measure radioactivity in a gamma counter should also be taken into account for the radiolabeling.

As a matter of fact, Karageorgou et al. in a recent study [167] assessed the in vivo behavior of ^68^Ga-Fe_3_O_4_-DPD MNPs as potential PET/MRI imaging agents. They demonstrated that the ex vivo biodistribution of the radionuclide mirrored the specific organ accumulation based on size and surface charge of Fe_3_O_4_-DPD MNPs. ^68^Ga-Fe_3_O_4_-DPD MNPs were distributed throughout the organs, while in the liver and spleen the uptake was the highest and the retention the longest at all time points examined, due to the presence of Kupffer cells which are able to recognize and engulf ^68^Ga-Fe_3_O_4_-DPD MNPs through phagocytosis. Moreover, the negative surface charge provided strong electrostatic repulsion between MNPs, resulting in excellent solubility and stability in the bloodstream with satisfactory blood retention at 30, 60, and 120 min post injection. The low lung uptake was attributed to possible embolization caused by post-injection aggregation (Figure 4).

Technetium-99m (^99m^Tc), indium-111 (^111^In), gallium-67 (^67^Ga), and iodine-125 (^125^I) are the most frequently used γ-emitting radionuclides for MNP radiolabeling. Of these, ^99m^Tc is the most common due to its favorable physical properties, such as its half-life, that allow for prolonged in vivo imaging and γ-photon single energy emission at 140 keV, which is beneficial for effective imaging.

Radiometals, both diagnostic (^64^Cu, ^68^Ga, and ^89^Zr) and therapeutic (^90^Y and ^177^Lu), are best attached to MNPs via chelation.^90^Y is a high energy β-emitter with optimal nuclear-physical characteristics (decay half-life 64.1h, Emax_β_ of 2.27 MeV) for radionuclide tumor therapy. Since labeling with different radionuclides most often requires new optimization of the procedure, ^90^Y is the radionuclide of choice for MNP-radiolabeling since it can be used for varied desirable purposes, from MNP tracking to the possible application in radionuclide therapy.

In recent years, the use of PET isotopes with a relatively long half-life, such as ^64^Cu (half-life 12.7h), and ^89^Zr (half-life 78.4 h), as well as the short-lived ^68^Ga (half-life 68 min), has increased. Especially the use of ^68^Ga (positron emission intensity 87%) is on the rise due to several identifiable properties of this radionuclide [168]. These include a superior image quality to that provided by SPECT radionuclides and the potential for on-demand production via a generator (^68^Ge/^68^Ga-generator).

### 6.4. Prediction of the Biodistribution Based on the Physicochemical Characteristics of MNPs

Systemically injected MNPs are rapidly cleared from the blood stream by the reticuloendothelial system mainly through the liver, spleen, and bone marrow, resulting in a low therapeutic index. Opsonization is the major factor that induces mononuclear phagocytic system (MPS) uptake of MNPs. Development of MNPs that avoid rapid clearance is a necessary requirement for sufficient delivery to the desired target. MNPs with an extremely high circulation half-life should also be avoided as this may contribute to off-target tissue toxicity and reduced signal-to-noise ratio due to non-specific binding.

### 6.5. Particle Size and Charge Influence Biodistribution

The hydrodynamic size of MNPs is one of the most important factors that determine their biodistribution kinetics. Particle size plays a key role in clearance of these materials from the body, with small particles (<10 nm) being cleared via the kidneys, and larger particles (>10 nm) being cleared through the liver and the MPS. MNPs that have a mean diameter of approximately 100 nm show prolonged blood circulation and a relatively low rate of MPS uptake. In the in vivo biodistribution study, the high tumor uptake exhibited by slightly negatively charged MNPs might be due to their prolonged blood circulation time. On the contrary, rapid clearance of highly charged MNPs by the RES resulted in relatively short half-life and low tumor uptake [169]. As MNPs are mainly eliminated by the phagocytic uptake in the RES and the liver is the main organ in the RES, the extent of sequestration of MNPs by the liver has been reported to correlate with the level of blood retention. The average particle size and polydispersity index of surface-modified MNPs can be determined by DLS.

### 6.6. The Nature of Coating Polymer Influences MNPs Pharmacokinetics

The surface characteristics of MNPs greatly influence their PK. Coating materials such as chitosan can sterically stabilize the corona and prevent aggregation of MNPs. Surface modification of MNPs with synthetic polymers like polyethylene glycol (PEG), polyvinyl alcohol (PVA), or polysaccharide can enhance solubility of hydrophobic materials, minimize non-specific binding, prolong circulation time, and enhance tumor-specific targeting. Generally, MNPs that have a mean diameter of approximately 100 nm with a neutral and hydrophilic polymer-extended surface exhibit prolonged blood circulation and an increased level of tumor delivery.

### 6.7. The Surface Charge of MNPs Modifies Their Biodistribution

The surface charge of MNPs depends directly on the molecular structure of the coating materials. The charge affects the degree of protein adsorption onto the MNPs surface. Due to charge-related adsorption by serum proteins, purely anionic or cationic charge increases the hydrodynamic diameter to >15 nm. According to literature data, liver uptake of charged particles is greater than that of neutral particles. Neutral MNPs exhibit a decreased rate of MPS uptake and prolonged blood circulation compared to charged ones. The zeta potential has been routinely used as a parameter for estimating the surface charge of MNPs.

### 6.8. Injected Quantity (Dose) of MNPs for Pharmacokinetic and Biodistribution Studies

The dose of MNPs is an important factor that can affect their PK and biological behavior. Therefore, the optimum doses of MNPs should be determined. Dose is expressed as weight of MNPs per unit weight of test animal (e.g., mg/Kg). Note, however, that in the case of radiolabeled MNPs the term “dose” may refer to the activity per unit weight (e.g., Bq/kg or Ci/kg), rather than their quantity.

Determination of the administered dose is necessary since the fate of certain MNPs in the body may differ according to the dose: urinary elimination is characteristic for low MNP concentration; transfer to the liver, biliary excretion and elimination in stools for moderate MNP concentration and finally liver storage in Kupffer cells (agglomerate and aggregate formation) is characteristic for high MNP concentration.

For i.v. administration, the maximum volume of liquid that can be administered as a single injection depends on the size of the animal used and route of administration. For mice and rats, a possible maximal i.v. administration volume that is considered good practice is 5 mL/Kg [170]. The essential requirement is to ensure that the volume given causes minimum discomfort and does not result in physiological or pathological changes that would compromise the experiment. Variability in test volumes should be minimized by adjusting the concentration to ensure a constant volume at all dose levels.

For intratumoral injection, up to 20% volume may be administered with limited leakage to the body, depending on tumor composition, density, vasculature and interstitial pressure. Immediately after injection, the application site should be covered and wiped twice with absorbing paper to retrieve any MNPs leaking out of the tumor.

### 6.9. Requirements for Animal Experimentation

Variation in animal models (e.g., mouse, rat, pig) and differences in MNP concentration compared to humans should be carried out. All experiments must be approved by the Institutional Animal Ethical Committee and performed in accordance to the guidelines of the local ethical committee. The request to the ethical committee should be sent as soon as possible since acquiring approval takes time, depending on the country, from one to several months. Animals should be maintained on standard pellet diet with free access to water in a 12-h light and dark cycle.

At least 3 animals should be used at each dose level and time point. However, power analysis should be carried out to statistically determine the optimum number of animals per group. Indeed, too small a sample size can miss the real effect in an experiment, whereas too large a sample size may lead to unnecessary wasting of resources and animals [171], and is not in accordance with the “3Rs” principle (Replacement/Reduction/Refinement) of animal experimentation. The animals should be of the same sex. If females are used, they should be non-pregnant. The weight variation in animals used in a test should not exceed ±20% of the mean weight, since this also affects biodistribution. Measurement of most biochemical parameters is traditionally performed under standardized conditions, which include overnight fasting, in order to achieve a more controllable metabolic environment and eliminate variations, thus achieving uniformity in the environment.

### 6.10. Route of Administration

Basic PK studies should be performed by i.v. administration of MNPs through the tail vein. It is important to know the plasma half-life of the injected MNPs (i.e., the time between injection and elimination from the blood) and utilize this data to tune the characteristics of the MNPs, based on the desired circulation time for each specific application.

Biodistribution of MNPs in the animals after intra-peritoneal route should be similar to that of i.v. administration; the highest concentrations of MNPs should be observed in the liver and the spleen. However, the blood flow also takes them to other organs, e.g., the lungs.

Intratumoral administration of MNPs is used to concentrate them in the tumor. However, a small quantity can also be found in other organs throughout the body, e.g., liver, lungs, lymph nodes, brain, spleen, which depends on leakage of the tumor vasculature.

### 6.11. Metabolism and Excretion

It is important to determine the nature of the degradation products of MNPs, along with their elimination mechanism and kinetics, especially as these metabolites may possess certain toxic effects. The desired clearance mechanism can be a factor in the design of MNPs. Radiotracer methods can be applied to follow excretion of MNPs measuring the radioactivity in the kidneys, bladder for hydrophilic based coated MNPs and in liver and intestines for hydrophobic MNPs.

Metabolite assessment in urine, blood and other organs can be performed at various time points after MNP administration, to determine whether the MNPs are metabolized after their administration to the animal [172].

- In urine: Urine is collected at the time of sacrifice and is centrifuged before High-performance liquid chromatography (HPLC) analysis;
- In serum: Blood is collected at the time of sacrifice and allowed to clot. After sample centrifugation (10 min, 2000× *g*), the supernatant is carefully removed, treated with twice the volume of cold ethanol and centrifuged once again. The supernatant is subjected to HPLC analysis;
- In other organs (and tumor, if relevant): The organ sample is cut into small pieces and homogenized, after addition of ice-cold Phosphate-buffered saline (PBS) solution. The resulting homogenate is centrifuged (15 min, 2000× *g*), filtered through a 0.2 μm filter and analyzed by HPLC.

### 6.12. Elemental Analysis by Inductively Coupled Plasma Mass Spectroscopy

An alternative to using the radiotracer method to measure tissue accumulation of MNPs is to use inductively coupled plasma mass spectroscopy (ICP-MS) to determine the amount of one or more elemental components in the delivery system, for comparison to the initial components present [173].

Digestion of the samples for ICP measurements should be performed in an Advanced Microwave Digestion System as follows: whole organs for biodistribution studies should be precisely weighed and mixed in quartz inserts with 5 mL nitric acid (HNO_3_) and then heated in a microwave. Digestion should be conducted for 20 min at a constant temperature of 200 °C, with a prior warm-up linearly over 10 min to 200 °C. After total mineralization and cooling to room temperature, and without filtration, the solutions should be diluted to a fixed volume in 10 mL volumetric flasks with ultra-pure water with a conductivity of 0.055 μS/cm. The content of Fe in the digested sample solutions can then be determined by ICP-MS.

## 7. Conclusions

Hyperthermia in combination with Radiation Therapy is already used in a limited number of hospitals and will probably play an important role in future cancer therapies. Unlike Radiation Therapy, however, which is a well-established treatment in cancer management, Hyperthermia needs to overcome several practical difficulties that have, so far, prevented it from realizing its full potential. Magnetic Hyperthermia, mediated by MNPs, is a novel approach that may be the answer to those problems. In this work, we have outlined the tests and considerations, after synthesis and characterization of the MNPs, that a researcher must address in order to perform pre-clinical testing. We have discussed in vitro evaluation of MNPs, in vitro evaluation of MNP-cell interactions, in vivo evaluation of the MNPs, Magnetic Hyperthermia combined with Radiation Therapy, and PK and biodistribution studies of MNPs. As MNP-mediated Hyperthermia is a new field that has not matured yet, we hope that this review will help establish guidelines and protocols that will lead to clinical application.

## Figures and Tables

**Table 1:** Brief presentation of recent studies concerning cytotoxicity of MNPs, reproduced from Patil et al. [71]. The references in the table are the references in Patil’s paper, where more information can be found.

Coating Agent	Types of IONPs	Diameter (nm)	Type of Cells	Dose	Incubation Time	Types of Assay	Brief Results	Ref.
Silica	Bare IONPs	10 ± 3	Human dermal fibroblasts (HDFs) and human fibrosarcoma (HT-1080) in DMEM media	200–1000 μg/mL	24 h	CCK-8 and LDH	APTMS-TEOS-Fe_3_O_4_ showed more cytotoxicity in terms of metabolic activity compared to other MNPs in HDFs. All MNPs induced LDH leakage in HDFs and HT-1080 cells.	[62]
	TEOS-IONPs	100–150						
	APTMS-TEOS-IONPs	100–150						
	Bare IONPs	10–50	Peripheral blood lymphocytes in RPMI media	1–100 μg/mL	2 and 24 h	Annexin V-FIT Capoptosis detection	No significant difference between treated and untreated lymphocytes for 2 and 24 h.	[104]
	VTES-TEOS-IONPs	10–50						
	APTES/VTES-TEOS-IONPs	10–50						
	Bare IONPs	150–200 L	L929 fibroblasts in DMEM media	15–1000 mg/L	24–72 h M	MTT	Silica coating reduced cell toxicity. Sulfhydryl modification improved cell-compatibility and haemocompatibility.	[105]
	TEOS-IONPs							
	DMSA-TEOS-IONPs							
	TEOS-IONPs	15–20	MCF-7 and HeLa cells in DMEM media	0–200 μg/mL	24 h M	MTT	MCF-7 and HeLa cells showed good biocompatibility at various concentrations.	[106]
PEG	PEG-IONPs	~30	Hela cells and C6 cells in DMEM media	0.01–1 mg/mL	12 h	MTT	Cell viability was not affected at the concentration of 1 mg/mL.	[107]
	PEG-IONPs	10–15	NIH/3T3 in DMEM	1.5 to 192 μM	24 and 48 h	MTT	PEG-IONPs showed good compatibility, 86% (24 h) and 67% (48 h) at 192 μM.	[108]
	Bare IONPs	10–13	Macrophages (mice) in RPMI media	100 μg/mL	1 h	MTT	No significant changes in viability after 1 h by all IONPs. Bare IONPs produced highest ROS compared to PEG and COOH-PEG-IONPs.	[109]
	PEG- IONPs	100						
	COOH-PEG-IONPs	100						
	PEG-550-IONPs	8–11	Bovine vascular smooth muscle cells (VSMCs) in DMEM media	100–1000 ppm	5–24 h	LIVE/DEAD viability/Cytotoxicity Kit	Dose dependent cytotoxic response was found. PEG-2K showed higher cell viability compared to PEG-10K at 100 ppm.	[110]
	PEG-2K-IONPs							
	PEG-5K-IONPs							
	PEG-10K-IONPs							
	PEPABC: IONPs	36 ± 5	Mouse brain endothelial cell line (bEnd.3) in DMEM media	0–10 mg/mL	30 h	Resazurin dye assay	No cell death reported after 30 h exposure at 10 mg/mL.	[110]
Dextran	Dextran-IONPs	200–250	Head and neck squamous cell carcinoma: tonsilla (UT-SCC-60A) and the metastasis (UT-SCC-60B) in DMEM media	0.2–1.8 mM	0–120 h	MTT, Annexin-V apoptosis detection assay	MTT: Decreased cell toxicity of dextran-IONPs compared to Resovist® Annexin-V-apoptosis: no changes in cell viability when cells were treated at the concentration of 1.8 mM.	[112]
	Dextran-IONPs	100	Mouse melanoma cells (B16) and Chinese hamster lung; fibroblast cells (V79) in DMEM media	0–400 μg/mL	24 h	MTT	Slight changes in the cell viability were noticed as compared to control.	[113]
	Dextran-IONPs 9	9.12 ± 1.46 L	L929 fibroblast cells	50–1000 μg/mL	24 h	MTT	Significant reduction in cell viability at 1 mg/mL. Cells were 90% viable at 0.75 mg/mL.	[114]
	DEAE-dextran-IONPs	27–50	Murine mesenchymal stem/stromal cell (MSC) in DMEM media	50 μg/mL	3 h	CCK-8	No significant changes I the cell viability were noticed.	[115]
	Bare Fe_2_O_3_	7	Human bone marrow mesenchymal stromal cells (hBMSCs) hBMSCs-1: age 12 years; hBMSCs-2: age 54 years in α-modified eagle media (α MEM)	15.4 g of iron/mL	72 h	WST-1	The study compared physicochemical properties of bare Fe_2_O_3_ and nanoparticles coated with different coating agents. hBMSCs-1: significant reduction in cell viability by PLL-Fe_2_O_3_and mannose-Fe_2_O_3_ NPs; hBMSCs-2: reduction in cell viability by all IONPs, mostly by uncoated-Fe_2_O_3_ and PLL-Fe O NPs.	[116]
	Endorem® (Fe_3_O_4_ coated with dextran)	5.5						
PLL	PLL-Fe2O3	5.5						
PLL-dextran	PLL-Endorem	5.6						
PDMAAm	PDMAAm-Fe_2_O_3_	7.5						
Mannose	Mannose-Fe_2_O_3_	7						
Mono-meric	IONPs-*R*_1_	6.5–7.5	Murine primary brain cells (primary microglia, primary hippocampal neurons, and neuron–glia co-cultures) in DMEM media	0.5, 1.5 or 3.0 mM	6–24 h	PI staining	Extended incubation and dose dependent cell death was observed by all IONPs except Ferumoxytol. Ferumoxytol surprisingly increased the number of viable cells. IONPs-*R*_1_, *R*_2_ and Ferucarbotran were quickly ingested by microglial cells compared to Ferumoxytol.	[117]
citrate layer	IONPs-*R*_2_	7.5–8.7						
Carboxy-dextran	Ferucarbotran (Resovist®)	60						
Carboxymeth-dextran	Ferumoxytol (Feraheme®)	30						
Chitosan	Bare IONPs 5	50-100	Human L-O_2_ hepatocytes in RPMI media	1.25–20 μg/mL	24 h	MTT	Bare IONPs showed more cytotoxicity compared to FAPLCS-IONPs in L-O_2_ hepatocytes.	[118]
	FAPLCS-IONPs 1	136.60 ± 3.90						
	Bare IONPs 1	18	Primary human osteoblast cells (SV40) in DMEM media	20–300 μg/mL	48 h	CCK-8	Decreased viability found when cells were treated with bare IONPs at 100 and 300 μg/mL.	[119]
	CS-IONPs	35						
	CS-IONPs	2–8	Cervical carcinoma cell lines (HeLa and SiHa)	0–1000 μg/mL	24 h	XTT	Bare and CS-IONPs showed reduction in cell viability by 5% and 2% respectively. SiHa cells showed 8% reduction in cell viability at 1000 μg/mL.	[120]
Carbon	Fe@C/C	5–140	Human (HTB140), murine (B16-F10) melanoma cells and human dermal fibroblasts (HDF) in DMEM	0.0001–100 μg/mL	24 h	MTT	Decreased cell viability in melanoma cells. Murine melanoma cells were more sensitive to bare IONPs than human cells. Fe@C-COOH and Fe@C-CH_2_CH_2_-COOH showed weaker response to cells, and 80–100% cells remained viable.	[121]

Abbreviations: TEOS: tetraethyl ortho silicate, APTMS: (3-aminopropyl) trimethoxysilane, PEG: polyethylene glycol, VTES: triethoxyvinylsilane, FITC: fluorescein isothiocyanate, PLL: poly-L-lysine, DMSA: meso-2,3-dimeraptosuccinic acid, XTT: (2,3-bis-(2-methoxy-4-nitro-5-sulfophenyl)-2H-tetrazolium-5-carboxanilide), PEG-CS-PTH NPs: parathyroid hormone (PTH 1−34) loaded PEGylated chitosan nanoparticles, PEG-(550,2K,5K,10K)-IONPs: IONPs coated with PEGs of varying chain length, FAPLCS: folate-conjugated N-palmitoyl chitosan micelles, DEAE-dextran-IONPs: diethylamino ethyl (DEAE)-Dextran coated IONPs, PEPABC: (poly(ethylene glycol)-poly(aspartate) block copolymers), CS-IONPs: chitosan coated IONPs, Fe@C/C: bare carbon encapsulated IONPs, Fe@C-COOH, Fe@C-CH_2_CH_2_-COOH: carboxylic acid modified IONPs.

## Abbreviations

AMF	Alternating Magnetic Field
ALP	Alkaline phosphatase
BrdU	5-bromo-2′-deoxyuridine
CFSE	CarboxyFluoresceinSuccinimidyl Ester
CT	Computed Tomography
DLS	Dynamic Light Scattering
γ-GT	Gamma-glutamyl transferase
HPLC	High-performance liquid chromatography
HSP	Heat Shock Proteins
HT	Hyperthermia
ICP-MS	Inductively Coupled Plasma Mass Spectroscopy
ID	Injected Dose
MNP	Magnetic Nanoparticle
MH	Magnetic Hyperthermia
MPS	Mononuclear Phagocytic System
MRI	Magnetic Resonance Imaging
MTT	3-(4,5-dimethylthiazolyl-2)-2,5-diphenyltetrazolium bromide
NP	Nanoparticle
PEG	Polyethylene glycol
PET	Positron Emission Tomography
PK	Pharmacokinetic
PI	Propidium Iodide
RES	Reticuloendothelial System
RT	Radiation Therapy
SGOT	Serum Glutamic-Oxaloacetic Transaminase
SPECT	Single-Photon Emission Computed Tomography
TEM	Transmission Electron Microscopy
X-ray	X-radiation
XTT	sodium 3′-[1-(phenylaminocarbonyl)-3,4-tetrazolium]-bis(4-methoxy-6-nitro) benzene sulfonic acid hydrate
